# Improving fresh‐cut fruit salad quality and longevity with chitosan coating enriched with poppy seed phenolics

**DOI:** 10.1002/fsn3.4040

**Published:** 2024-02-20

**Authors:** Bahar Demircan, Yakup Sedat Velioglu

**Affiliations:** ^1^ Department of Food Engineering Ankara University Ankara Turkey

**Keywords:** edible coating, food preservation, fresh‐cut, fruit salad, microbial safety, phenolics, poppy seed, sensory evaluation, shelf life

## Abstract

This innovative study introduces the application of a 5% (v/v) poppy seed phenolic extract‐infused edible chitosan coating on fresh‐cut fruit salads (comprising apple, pineapple, pomegranate, and kiwi) stored at +4°C for 12 days. Non‐coated samples experienced notable changes: 4.30% weight loss, 25% decay, pH level at 3.59, titratable acidity of 0.18%, and browning index of 1.71. In contrast, fruit salads coated with chitosan–poppy seed phenolic extract exhibited significant improvements: weight loss reduced to 3.10%, decay limited to 3.13%, pH increased to 3.76, titratable acidity enhanced to 0.20%, and browning index notably decreased to 0.33. Soluble solids ranged from 11.83 to 14.71, L* from −8.13 to 18.64, a* from −1.85 to 22.35, and b* from 8.26 to 27.89 in non‐coated salads. Adding poppy seed phenolic extract to the coated fruits slightly expanded these ranges. Sensory evaluations consistently rated non‐coated samples between 1 and 3, while the coated samples received higher ratings between 6 and 7. These assessments consistently highlighted enhanced attributes, including intensified aroma, enriched color, improved taste, texture, and overall acceptability. Moreover, incorporating poppy seed phenolic extract amplified sensory qualities and significantly improved microbial safety (<10^6^ CFU/g). In summary, the chitosan‐based coating, enriched with poppy seed phenolic extract, effectively extended the shelf life of fresh‐cut fruit salads. This integrated approach preserves key attributes, ensures microbial quality, and enhances the sensory characteristics of these products. The study's results emphasize its potential as a pivotal innovation in food preservation by providing specific and tangible outcomes.

## Graphical abstract

This study presents a novel approach using a chitosan coating enriched with poppy seed phenolic extract to extend the shelf life of fresh‐cut fruit salads. Non‐coated samples exhibited significant decay, while coated samples showed reduced weight loss, enhanced pH levels, and improved sensory attributes, highlighting the potential of this innovative method for food preservation.

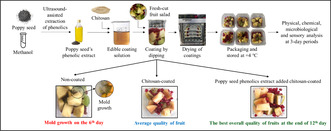

## 1 INTRODUCTION

Fresh‐cut products refer to food items, typically fruits or vegetables, which undergo a series of processes, including peeling, removing seeds if applicable, chopping, and packaging. These processes facilitate the provision of ready‐to‐eat products to consumers and minimize the need for further handling during storing and transporting fruits and vegetables. However, it is essential to note that these procedures often remove the natural epidermal layer of fruits and vegetables, increasing the risk of microbial contamination (Maringgal et al., 2020). Among the most common preservation methods for fresh‐cut fruits are cold storage, modified atmosphere packaging, or the addition of synthetic preservatives. However, consumers are increasingly conscious of health and food safety, leading to a preference for purchasing fresh‐cut fruits that do not contain synthetic additives (Lacivita et al., 2021). This is where the role of edible coatings comes into play. Edible coatings represent an innovative technology that enhances the fresh‐cut products' quality and shelf life. These coatings create a thin protective layer on the surfaces of fruits and vegetables, effectively shielding them from external factors, thereby preserving their freshness and preventing microbial growth. Furthermore, these coatings mitigate a significant issue in fresh‐cut fruits: the browning caused by oxidation (Kumar et al., 2018; Vishwasrao & Ananthanarayan, 2017). As a result, these coatings improve the quality of fresh‐cut products. In this way, fresh‐cut products can maintain their freshness for an extended period, prolonging their shelf life and offering consumers healthier and more nutritious options.

In this context, incorporating plant extracts into edible coatings has positively affected fruits and vegetables. First, in terms of reducing weight loss, coatings containing plant extracts can limit water loss, thus preventing the weight loss of fruits. Concerning firmness, these coatings can help preserve fruit texture, extending their freshness over a longer period. Regarding respiration rate and ethylene production, coatings enriched with plant extracts can regulate fruit metabolism, thus extending shelf life. Second, in terms of sensory quality, these coatings can maintain the taste, color, aroma, flavor, and texture of fruits, enhancing consumer satisfaction. Lastly, in terms of decay incidence or spoilage, coatings with plant extracts can restrict microbial growth on fruits, thereby reducing decay rates. Therefore, adding plant extracts to edible coatings has the potential to extend the shelf life and improve the quality of fresh‐cut fruits (Anjum et al., 2020; Nair et al., 2020; Ozdemir & Gokmen, 2017; Tesfay & Magwaza, 2017). In this context, studies applying coatings with added plant extracts to fresh‐cut fruits are standard in the literature. However, there needs to be more research examining the extension of the shelf life of fruit salads, which are becoming increasingly popular with edible coatings.

The limited shelf life of fresh‐cut fruit salads, typically 1 to 3 days, poses significant economic and social challenges (Lacivita et al., 2021). As consumer demand for these products continues to grow, there is a pressing need to effectively extend their shelf life while maintaining high quality and safety standards. This study uses edible coatings enriched with plant extracts to address this issue. In this context, particular attention is given to poppy (*Papaver somniferum* L.) seed extract, a relatively understudied area in the literature. While various plant extracts have been incorporated into different polymer matrices to enhance coating properties (Adilah et al., 2018), the phenolic extract from poppy seeds, renowned for its valuable oil content, remains an unexplored avenue in this research field.

Poppy is a historically significant plant cultivated globally for medicinal and culinary purposes. Its alkaloids, oils, and proteins have made it a crucial component in various industries, including medicine, food, cosmetics, and paint. Poppy seeds contain approximately 50% oil, 25% protein, 5% moisture, 5% fiber, and 15% vitamins and minerals. While research on poppy seed oil is extensive, investigations into its phenolic content are limited. Phenolic compounds found in different parts of the poppy plant exhibit antioxidant properties with various biological advantages. Due to their fatty acids and phenolic compounds, poppy seeds demonstrate substantial antioxidant potential, as evidenced by their impressive ability to scavenge free radicals (Pushpangadan et al., 2012).

This study aims to investigate the potential of chitosan‐based coatings enriched with blue poppy seed phenolic extract to extend the shelf life of fresh‐cut fruit salads. While prior research has explored edible coatings with plant extracts to preserve fresh‐cut fruits, this work narrows its focus to the fruit salad market segment, which is rapidly growing and increasingly popular. This study introduces an innovative approach to enhance fresh‐cut fruit salads' quality, safety, and longevity by incorporating phenolic extract from blue poppy seeds. Furthermore, it ventures into the relatively unexplored realm of utilizing poppy seed extracts to improve coating properties, opening a promising avenue for future food preservation and quality enhancement.

## 2 MATERIALS AND METHODS

Apples (*Malus domestica*), pomegranates (*Punica granatum*), kiwis (*Actinidia chinensis*), and pineapples (*Ananas comosus*) were sourced from a local market in Ankara, Turkey, for the creation of the fresh‐cut fruit salad. Only fruits in pristine condition, uniform size, regular shape, and commercial maturity, without any defects, were selected. Approximately 2 kg of each fruit type was used for the experiment. After thorough washing, the apples, kiwis, and pineapples were peeled and cut into pieces measuring around 2 × 2 cm using a stainless‐steel knife. The pomegranates were granulated. The chitosan (low molecular weight, CAS No.: 9012‐76‐4) and all other chemicals used in the study were obtained from Sigma‐Aldrich and Merck.

**FIGURE 1:**
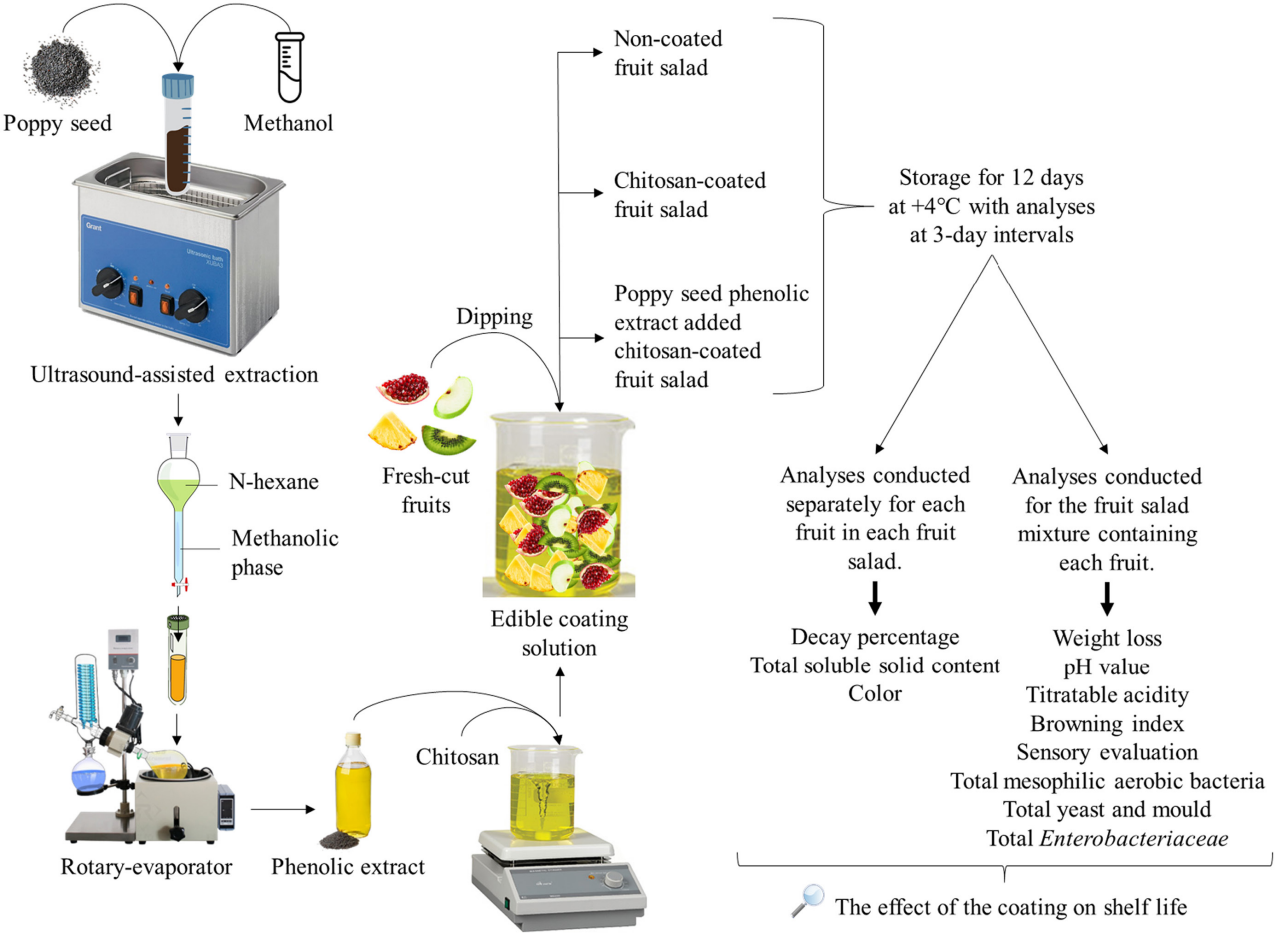
Schematic diagram of the research steps.

All experiments were conducted in triplicate and three parallel runs and a schematic representation of the research steps is provided in Figure 1.

### 2.1 Extraction of phenolics from poppy seeds

To extract phenolic compounds from poppy seeds, 5 g of finely ground seeds was combined with 15 mL of methanol and subjected to ultrasonic‐assisted extraction (SK 3310 LHC, China) at 35 kHz frequency, 100% power, and 25°C for 60 min. After extraction, the samples underwent centrifugation at 5000 rpm for 10 min (Hettich Zentrifugen Universal 320R, Germany). After removing the supernatant, the remaining residue underwent a second extraction with methanol under identical conditions. Subsequently, 10 mL of N‐hexane was mixed and vortexed (Heidolph D‐91126, Germany) with the supernatants, and then the methanol and N‐hexane layers were separated using a separatory funnel. This process was repeated twice. The resultant phenolic phase was concentrated by drying at 50°C using a rotary evaporator (Buchi R100, Switzerland), and the residue was reconstituted with 1.5 mL of ethanol before utilization (Ghafoor et al., 2019).

### 2.2 Edible coating to fresh‐cut fruit salad

The fruit salad, composed of equal amounts of each fruit group, was prepared in 100 g portions. The edible coating solution was created by blending 2% (w/v) chitosan in a 1% (v/v) acetic acid solution for 4 h using a magnetic stirrer (Heidolph MR Hei‐Standard, Germany). Subsequently, 1% (w/v) glycerol and 5% (v/v) poppy seed phenolic extract (determined through preliminary experiments) were added, and the mixture was homogenized at 13,500 rpm for 5 min using an Ultra‐Turrax mixer (Heidolph Silent Crusher M, Germany). The fruit salad was coated by immersing 100 g of the mixture in 100 mL of the coating solution for 30 min and then draining the excess solution for 5 min. The coated samples were then air‐dried at 24°C for 30 min using an oven (Memmert 100‐800, Germany) to solidify the edible coating on the surface. Then, the samples were placed in plastic containers with lids. The sample groups included non‐coated (NC), chitosan‐coated (CH), and poppy seed phenolic extract added chitosan‐coated (CHP). These samples were stored at +4°C for 12 days and were subjected to analysis at 3‐day intervals (Demircan & Ozdestan, 2021a).

### 2.3 Weight loss

Weight loss (%) was determined gravimetrically for each storage period (Naeem et al., 2018).

### 2.4 Decay percentage

The percentage of decay (%) in fruit salads was calculated following the method described by Riaz et al. (2021), wherein 10 fruits from each treatment were selected for analysis.

### 2.5 pH, titratable acidity, and browning index

For the analysis, 10 g of fruit salad (2.5 g from each fruit) was homogenized with 50 mL of distilled water, followed by centrifugation at 5000 rpm for 5 min at 20°C. The resulting supernatant was filtered through filter paper. The pH of the filtrate was measured at room temperature using a digital pH meter (WTW InoLab pH 720, Germany). For titratable acidity determination, 1 mL of the filtrate was diluted with 4 mL of distilled water and titrated against 0.1 N NaOH with phenolphthalein as an indicator. Titratable acidity (%) was calculated in terms of malic acid. The browning index was determined by directly measuring the absorbance of the filtrate at 420 nm using a ultraviolet–visible (UV‐Vis) spectrophotometer (Shimadzu UV‐1601, Japan), following the method outlined by Sobral et al. (2017).

### 2.6 The total water‐soluble solids content

The water‐soluble dry matter content (%) of each fruit in the fruit salad was determined separately as °Brix using a refractometer (Atago Rx‐5000a, USA), following the method described by Naeem et al. (2018).

### 2.7 Color

The surface colors (L*, a*, b*) of each constituent fruit in the fruit salad were individually measured using a color measuring device (Konica Minolta CR‐400, Japan), following the procedure outlined by Demircan and Ozdestan (2021b).

### 2.8 Sensory evaluation

The sensory attributes of the fruit salads, including appearance, aroma, texture, taste, and overall acceptability, were assessed by a panel of 12 semi‐trained evaluators. The assessment was conducted using a 9‐point hedonic scale (1: poor to 9: excellent) within a dedicated sensory analysis facility.

### 2.9 Microbiological analysis

For microbial analysis, 10 g of the fruit salad (2.5 g from each fruit) was homogenized (MIX 2, AES Laboratoire, France) with 90 mL of a saline solution (0.85% w/v sodium chloride (NaCl)). The mixture was then appropriately diluted to achieve the desired microbial count. From these dilutions, samples were plated on specific agar media: Plate Count Agar (PCA, Merck) for total mesophilic aerobic bacteria, Yeast Glucose Agar (YGC, Merck) for total yeast and mold, and Violet Red Bile Glucose Agar (VRBG, Merck) for total *Enterobacteriaceae* count. After incubation at 30°C for 48 h, 25°C for 3–5 days, and 37°C for 24 h, respectively, the results were reported as log colony‐forming unit (CFU)/g, following the methodology outlined by Lacivita et al. (2021).

### 2.10 Statistical analysis

Statistical analysis of all the obtained results was conducted using ANOVA (analysis of variance). Duncan's test was employed for comparisons, and significance was determined at a threshold of *p* < .05. All statistical analyses were performed using SPSS 22.0 software (SPSS Inc., USA).

## 3 RESULTS

### 3.1 Weight loss

Water loss, mainly caused by moisture loss, significantly deteriorates the quality of fresh‐cut fruits, making them more prone to postharvest issues and resulting in economic losses (Yousuf et al., 2021). These reductions contribute to economic losses and impact the quality of shelf life (Karagoz & Demirdoven, 2019). The weight losses of the different sample groups over the storage duration are presented in Table 1. The weight loss demonstrated an escalating trend across all sample groups throughout the storage intervals. The presence of edible coatings on the fruit surface effectively inhibits water loss from the fruit structure, thereby mitigating weight loss (Firdous et al., 2022; Pham et al., 2023). The inherent moisture‐resistant properties of chitosan coatings are further bolstered by including poppy seed phenolic extract, contributing to their exceptional performance. This improvement can be attributed to the synergistic effect of chitosan and the phenolic extract, which form an enhanced protective shield against moisture loss, ultimately reducing weight loss. Similarly, Riaz et al. (2021) highlighted the effectiveness of chitosan‐based edible coatings enriched with apple peel polyphenols in reducing weight loss in strawberries and preserving their quality, emphasizing the positive role of natural compounds in enhancing coating performance.

**TABLE 1:** Change in chemical quality parameters of fruit salads during storage.

	Period (day)	NC	CH	CHP
Weight loss (%)	0	0^c^	0^e^	0^e^
	3	0.77 ± 0.12^b^	0.75 ± 0.21^d^	0.70 ± 0.07^d^
	6	4.30 ± 0.08^a^	2.07 ± 0.30^c^	1.87 ± 0.22^c^
	9	ND	3.15 ± 0.17^b^	2.93 ± 0.30^b^
	12	ND	4.60 ± 0.08^a^	3.10 ± 0.16^a^
pH	0	3.71 ± 0.03^a^	3.69 ± 0.03^d^	3.70 ± 0.01^c^
	3	3.59 ± 0.01^b^	3.68 ± 0.01^d^	3.68 ± 0.01^d^
	6	3.59 ± 0^b^	3.73 ± 0.01^b^	3.72 ± 0.01^b^
	9	ND	3.75±0^a^	3.76 ± 0.01^a^
	12	ND	3.71 ± 0.01^c^	3.76±0^a^
Titratable acidity (%)	0	0.29 ± 0.38^a^	0.16 ± 0.35^c^	0.16 ± 0.27^d^
	3	0.15 ± 0.18^c^	0.20 ± 0.67^ab^	0.22 ± 0.30^a^
	6	0.18 ± 0.08^b^	0.22 ± 0.22^a^	0.20 ± 0^ab^
	9	ND	0.16 ± 0.17^c^	0.18 ± 0.34^c^
	12	ND	0.18 ± 0.07^bc^	0.20 ± 0.09^b^
Browning index (abs)	0	0.51 ± 0.01^c^	0.10 ± 0.02^d^	0.10 ± 0.03^d^
	3	0.67 ± 0.05^b^	0.32 ± 0.04^c^	0.27 ± 0.04^c^
	6	1.71 ± 0.10^a^	0.39 ± 0.07^b^	0.29 ± 0.04^b^
	9	ND	0.42 ± 0.12^a^	0.30 ± 0.11^b^
	12	ND	0.43 ± 0.80^a^	0.33 ± 0.07^a^

*Note*: Different superscripts in the same column on the basis of analysis represent differences between sample groups according to Duncan's test (*p* < .05).

Abbreviations: CH, chitosan‐coated: CHP, poppy seed extract added chitosan‐coated; NC, non‐coated; ND, analysis not performed because storage was terminated.

Numerous researchers have illustrated the capacity of edible coatings enriched with diverse components to diminish weight loss in fruits (Amiri et al., 2021; Farina et al., 2020; Ghosh et al., 2021; Treviño‐Garza et al., 2017). Kumar, Baswal, et al. (2021) showcased this effect on guava fruit, while Hajebi Seyed et al. (2021) and Kumar, Petkoska, et al. (2021) yielded similar results for mango fruit. Saleem et al. (2021) and Khodaei et al. (2021) reported similar outcomes for strawberries. Carbone et al. (2021) emphasized that edible coatings enriched with *Humulus lupulus* cone extract significantly reduced weight loss during the storage of fresh‐cut kiwis. Du et al. (2023) and Aayush et al. (2022) confirmed that phytochemical‐based edible coatings for fresh‐cut apples effectively curtailed weight loss, extending the shelf life while maintaining fruit quality. These collective findings underscore the remarkable efficacy of edible coatings in reducing weight loss in various fresh‐cut products. Pleșoianu and Nour (2022) further validated this by concluding that enriched pectin‐based edible coatings reduce weight loss while preserving pear quality in fresh‐cut pears. Saleem et al. (2022) reported a significant reduction in weight loss in date fruits upon applying hydrocolloid‐based edible coatings.

In summary, applying edible coatings enriched with natural compounds, such as poppy seed phenolic extract, proves highly effective in minimizing weight loss in various fresh‐cut fruits. This preservation strategy significantly contributes to extending the shelf life and upholding fruit quality, demonstrating its potential as a valuable tool in the fresh‐cut produce industry.

### 3.2 pH levels and Titratable acidity

Titratable acidity and pH levels are fundamental parameters with direct implications for the sensory characteristics of foods (Mojaddar Langroodi et al., 2021). pH plays a pivotal role in influencing various chemical processes critical to the qualities of fresh‐cut fruits, including the Maillard reaction, enzymatic activity, and microorganism growth. Our observations in Table 1 reveal changes in pH values throughout storage, indicating a decrease in pH for NC samples and an increase for CH and CHP samples from the first to the final day of storage.

The fluctuations in the pH levels in fruit salads are likely the results of shifts in fruit biochemical activity during storage, variations in respiration rate, ongoing metabolic processes, and the breakdown of acids through respiration. The decline in pH values in NC samples might be attributed to the generation of organic acids from carbohydrate breakdown or the accumulation of metabolites due to microbial proliferation. In contrast, CH and CHP samples, featuring the application of the edible coatings, effectively decelerated the metabolic activity of the fruits, thus postponing the respiration process. As a consequence, substrates like organic acids underwent less degradation and similar documented pH alterations in the literature, including studies on fruit salads (Martins et al., 2016), pineapple (Rodríguez et al., 2021), apple (Madanipour et al., 2019), pomegranate (Amiri et al., 2021), and kiwifruit (Huang et al., 2017).

Titratable acidity, reflecting the concentration of organic acids in fruits, exhibits a declining trend associated with the depletion of these acids, thus serving as a marker of fruit aging. The values of titratable acidity, presented in Table 1, show that the NC samples experience a decrease in acidity by the end of the storage period due to the utilization of organic acids in the respiration process. The decline in acidity during ripening results from converting acids into sugars and the heightened utilization of fruit components in metabolic activities. Contrasting these findings, the CH and CHP sample analysis demonstrates that titratable acidity undergoes controlled and marginal changes. This is credited to the chitosan coating's effectiveness in slowing down the fruit's metabolic rate, in line with analogous results in the existing literature (Amiri et al., 2021; Karagoz & Demirdoven, 2019; Martins et al., 2016; Rodríguez et al., 2021).

In our investigation, we have ascertained that adding poppy seed phenolic extract to chitosan, denoted as CHP, exhibited superior effectiveness in preserving pH and titratable acidity levels in fresh‐cut fruits. The performance of CHP can be attributed to the synergistic effect of chitosan and the phenolic extract derived from poppy seeds. Chitosan, a natural biopolymer, forms a semipermeable barrier when applied as an edible coating. This barrier limits the exchange of gases between the fruit and its surroundings and effectively slows down the respiration rate of the fruit. Consequently, the degradation of organic acids, which contribute to titratable acidity, is considerably reduced. Poppy seed phenolic extract, rich in antioxidant compounds, further enhances this protective shield (Saeed et al., 2022). These antioxidants scavenge free radicals and mitigate the oxidative processes that often lead to the breakdown of organic acids (Melo et al., 2022). Thus, the combined action of chitosan and poppy seed phenolic extract provides a robust defense against the degradation of pH and titratable acidity during storage. Our research resonates with the findings presented by Radi et al. (2023), who observed substantial shifts in pH and titratable acidity levels in tangerine fruits during storage, signifying an upward trend in pH alongside a decline in acidity. This trend mirrors the intricate mechanisms involved in altering pH and titratable acidity. Moreover, Sicari et al. (2017) contributed to this understanding by linking the elevation in pH values and the reduction in acidity to the transformation of organic acids into sugars during the ripening of fruits. Consequently, our findings emphasize the potential of incorporating poppy seed phenolic extract into chitosan‐based edible coatings as a valuable strategy for enhancing the postharvest quality of fresh‐cut fruits.

### 3.3 Browning index

The undesirable browning phenomenon is the primary challenge impacting fresh‐cut fruits' shelf life. This browning results from enzymatic processes catalyzed by polyphenol oxidases (PPOs) and leads to the formation of brown coloration, notably in polyphenol‐rich fruits like apples. Apples, along with other fruits such as pineapples, melons, grapes, and pears, are commonly utilized in fresh‐cut production and are susceptible to such enzymatic browning reactions (Leneveu‐Jenvrin et al., 2020; Najafi Marghmaleki et al., 2021).

The increase in the browning index across all samples during storage primarily stems from the browning of light‐colored fruits such as apples and pineapples. This phenomenon is attributed mainly to enzymatic browning (Miteluț et al., 2021). Enzymatic browning diminishes the visual appeal of fresh‐cut fruits and leads to undesirable alterations in their flavor profile.

The browning indices for all sample groups are outlined in Table 1. Initially, the browning index values were relatively low across all groups, with NC having the highest value. At the same time, both CH and CHP displayed lower values, indicating minimal browning at the beginning of the storage period. However, by the 3rd day, an increase in the browning index values was observed in all groups, signifying the onset of enzymatic reactions leading to browning. Notably, the NC group experienced the most significant increase in browning. As the storage duration extended to the 6th day, the NC group displayed the highest browning index value, indicating a substantial increase in browning. In contrast, the CH and CHP groups also exhibited increased browning index values, albeit notably lower than the NC group. Unfortunately, data are unavailable (ND) for the NC group on days 9 and 12, while both CH and CHP groups showed only a slight increase in their browning index values. These results imply that, although ongoing, browning remained relatively high beyond day 9. This suggests that the coatings (CH and CHP) effectively slowed down the browning process during the later stages of the storage period.

The enhanced effectiveness of the CHP group in reducing the browning index can be attributed to the inclusion of the poppy phenolic extract in the edible coating. Despite the coating's antioxidative properties, its phenolic compounds can also serve as substrates for PPO activity, potentially contributing to the browning of the cut fruit surfaces, as observed in previous studies. Notably, the study by Kumar, Ojha, et al. (2021) highlighted how the chitosan–pullulan coating with pomegranate peel extract effectively mitigated browning in bell pepper. This outcome suggests that the poppy phenolic extract in the CHP coating played a crucial role in reducing the browning index, possibly by inhibiting PPO activity or through antioxidant mechanisms that counteract the browning reactions.

By combining the phenolic extract's antioxidant properties with the coating's protective barrier, the CHP group exhibited superior performance in minimizing browning. This synergy between the phenolic extract and the coating matrix likely enhanced the overall effectiveness in reducing browning, especially in the later storage stages.

In a similar vein, Sobral et al. (2017) reported a browning index of 0.53 for a fruit salad composed of apple, melon, mango, and grape, as well as a browning index of 0.90 at 420 nm for a fruit salad containing orange, kiwi, pineapple, and mango, where antioxidant compounds were employed. Furthermore, Najafi Marghmaleki et al. (2021) demonstrated that using alginate coating and anti‐browning agents like citric acid and ascorbic acid effectively prevented browning in freshly cut apples. They substantiated this phenomenon with high L values and low browning index values. Lara et al. (2020) reported reduced enzymatic browning in fresh‐cut lotus roots when coated with xanthan gum. Likewise, Vishwasrao and Ananthanarayan (2017) observed a positive effect of methyl cellulose‐based coating on the prevention of browning in sapota fruits. Passafiume et al. (2020) conveyed how composite coatings comprising aloe vera gel, hydroxypropyl methylcellulose, and lemon essential oil effectively reduced browning in fresh‐cut kiwis. These examples collectively emphasize the diverse strategies employed in previous research to combat browning in various fruits and vegetables. This aligns with our study's observations regarding the CHP coating's enhanced performance due to the poppy phenolic extract's antioxidant properties.

### 3.4 Decay percentage

**FIGURE 2:**
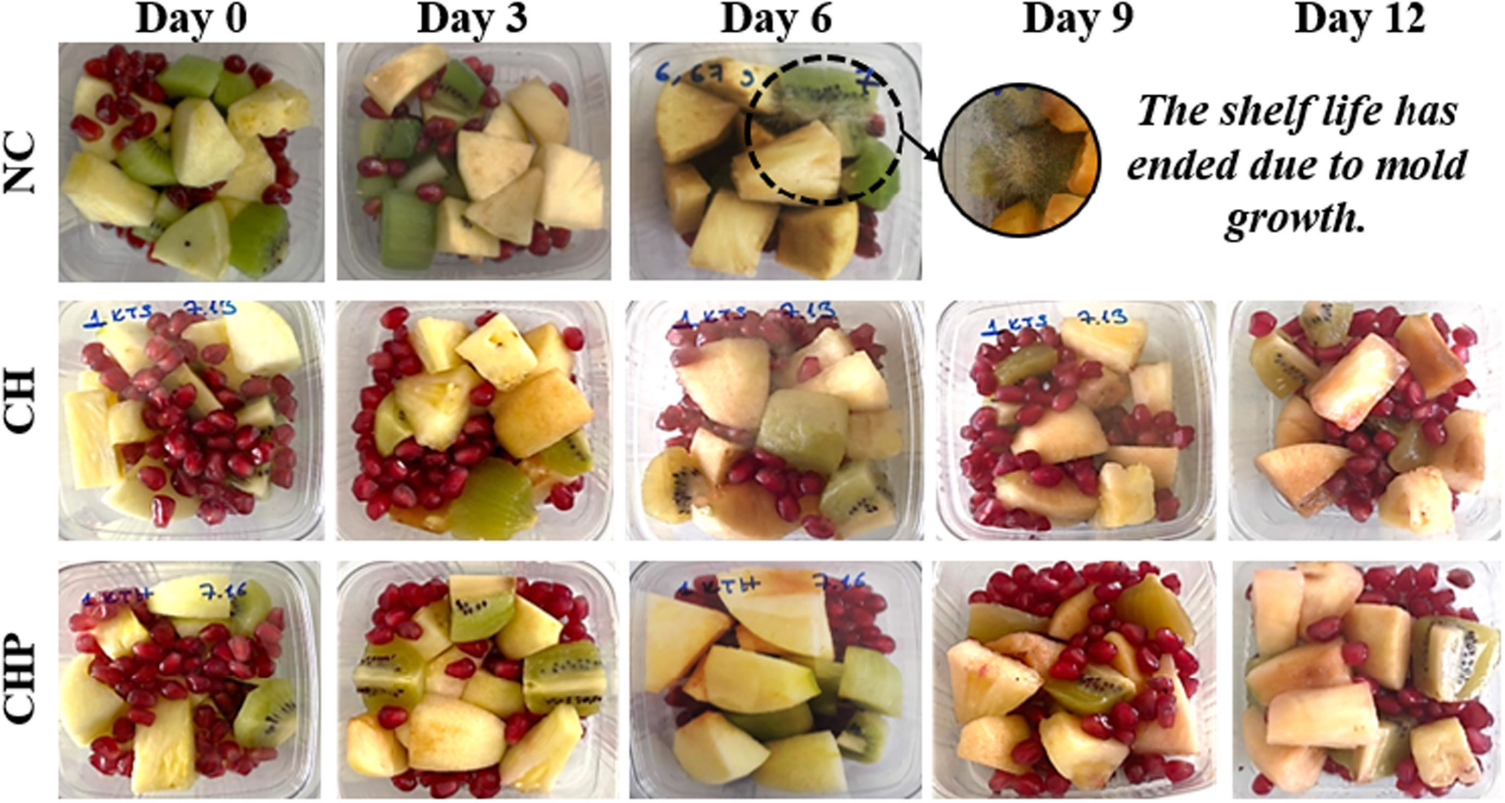
The images of sample groups (CH, chitosan‐coated; CHP, poppy seed phenolic extract added chitosan‐coated; NC, non‐coated) throughout storage.

The decay percentage in fresh‐cut fruits is assessed through physical observation, considering aspects, such as softening, deterioration, and browning. Inadequate postharvest storage conditions or delayed consumption can accelerate the decay rate. When applied to fruits, edible coatings safeguard their quality, aroma, moisture content, and color postharvest. The decay percentages presented in Table 2 were calculated based on observations of 10 fruits from each fruit group. The images of sample groups throughout storage are presented in Figure 2.

**TABLE 2:** Decay percentages (%) of each fruit group in fruit salads.

	Period (day)	NC	CH	CHP
Pineapple	0	0^c^	0^b^	0^b^
	3	5 ± 1.15^b^	0^b^	0^b^
	6	25 ± 1.53^a^	3 ± 0.66^a^	0^b^
	9	ND	3 ± 0.81^a^	3 ± 0.19^a^
	12	ND	5 ± 0.40^a^	3 ± 0.87^a^
Apple	0	0^c^	0^e^	0^e^
	3	10 ± 1.21^b^	5 ± 0.73^d^	5 ± 0.67^d^
	6	25 ± 0.47^a^	10 ± 0.63^c^	8 ± 0.70^c^
	9	ND	15 ± 0.40^b^	10 ± 1.17^b^
	12	ND	18 ± 1.51^a^	13 ± 1.68^a^
Kiwi	0	0^e^	0^c^	0^b^
	3	5 ± 1.06^b^	0^c^	0^b^
	6	25 ± 1.40^a^	0^c^	0^b^
	9	ND	2 ± 0.73^b^	0^b^
	12	ND	4 ± 0.33^a^	3 ± 0.63^a^
Pomegranate	0	0^f^	0^c^	0^b^
	3	0^f^	0^c^	0^b^
	6	25 ± 0.19^a^	0^c^	0^b^
	9	ND	5 ± 1.81^b^	2 ± 0.89^b^
	12	ND	15 ± 0.41^a^	10 ± 1.07^a^

*Note*: Different superscripts in the same column on the basis of fruit represent differences between sample groups according to Duncan's test (*p* < .05).

Abbreviations: CH, chitosan‐coated: CHP, poppy seed extract added chitosan‐coated; NC, non‐coated; ND, analysis not performed because storage was terminated.

According to the results, the decay percentage increased with the storage duration for pineapple, apple, kiwi, and pomegranate fruits. The initial decay percentage of these fruits was the highest in the NC (control) group, while it was lower in the CH and CHP groups. Particularly on the 6th day of storage, a substantial decay was observed in the NC group's pineapple, apple, and kiwi fruits. In contrast, the decay rate significantly decreased in the CH and CHP groups. Mold growth was noticed in the NC samples from the 6th day onward, exceeding the acceptable threshold for fruit decay, a maximum of 25% (Zarbakhsh et al., 2019), leading to the termination of storage for the NC samples on the 6th day.

These results indicate that the CHP coating effectively controlled fruit decay. Adding poppy phenolic extract to the CHP coating enhanced its antioxidant properties and reduced decay. These findings emphasize the potential of developing edible coatings for prolonged preservation of fruits during storage.

The relatively slower decay rate in coated fruits can be attributed to the protective shield formed by the edible coatings on the fruit surface, effectively mitigating the influence of external factors, particularly oxygen contact. Similar findings have been reported in the literature. Hashemi and Jafarpour (2021) demonstrated a konjac glucomannan‐based coating, and Manzoor et al. (2021) showed that nanoemulsion coatings reduced decay percentages in fresh‐cut kiwis. Khodaei et al. (2021) and Saleem et al. (2021) observed that coated strawberries exhibited a significantly slower decay rate than control samples.

### 3.5 The total water‐soluble solids content

Sugars constitute the predominant component of water‐soluble dry matter in fresh fruits. While immature fruits' average water‐soluble dry matter content is approximately 11.4 °Brix, it rises to above 13.9 °Brix for fully ripe fruits (Ghosh et al., 2021). Preserving total water‐soluble solids is essential for maintaining fruit quality and flavor over an extended period (Suriati, 2021).

According to our study, °Brix values of fruits exhibited variations across different groups during the storage period (Table 3). Initially, in the case of pineapple fruits, the NC group displayed the highest °Brix values, while the CH and CHP groups had lower values. However, by the 6th day, we observed an increase in °Brix values in all groups, except in the NC group. A significant increase was observed in the CH and CHP groups, starting from the 3rd day. Similar trends were noticed in apple fruits, with the NC group having initially higher °Brix values than other groups. However, by the 6th day, the CH and CHP groups surpassed the NC group regarding °Brix values. This pattern was also observed in kiwi fruits, with a consistent increase in °Brix values throughout the storage period. For pomegranate fruits, °Brix values increased in all groups as the storage period advanced. The overall rise in °Brix values over time signifies an increase in the sugar content of the fruit samples.

**TABLE 3:** °Brix values (%) of each fruit group in fruit salads.

	Period (day)	NC	CH	CHP
Pineapple	0	12.54 ± 0.21^c^	12.58 ± 0.58^d^	13.09 ± 0.49^c^
	3	12.73 ± 0.23^b^	13.91 ± 0.08^c^	14.84 ± 0.48^a^
	6	12.89 ± 0.49^a^	13.98 ± 0.21^b^	14.42 ± 0.57^a^
	9	ND	14.29 ± 0.04^a^	13.89 ± 0.06^b^
	12	ND	14.22 ± 0.82^a^	14.57 ± 0.54^a^
Apple	0	13.17 ± 0.18^a^	12.13 ± 0.55^e^	14.72 ± 0.35^c^
	3	14.12 ± 0.66^a^	12.90 ± 0.51^d^	14.23 ± 0.15^d^
	6	14.71 ± 0.13^a^	14.46 ± 0.63^c^	15.12 ± 0.58^a^
	9	ND	15.59 ± 0.24^a^	13.54 ± 0.31^e^
	12	ND	14.72 ± 0.19^b^	14.96 ± 0.18^b^
Kiwi	0	11.30 ± 0.04^b^	13.41 ± 0.11^d^	13.05 ± 0.26^d^
	3	9.81 ± 0.27^c^	13.28 ± 0.41^e^	13.08 ± 0.24^c^
	6	11.83 ± 0.07^a^	13.88 ± 0.22^c^	13.33 ± 0.05^b^
	9	ND	14.73 ± 0.18^a^	13.70 ± 0.41^a^
	12	ND	14.02 ± 0.21^b^	13.71 ± 0.28^a^
Pomegranate	0	15.65 ± 0.52^a^	16.07 ± 0.25^a^	16.54 ± 0.22^a^
	3	13.85 ± 0.65^c^	15.79 ± 0.31^b^	15.87 ± 0.54^b^
	6	14.40 ± 0.77^b^	14.92 ± 0.48^c^	15.56 ± 0.44^c^
	9	ND	14.68 ± 0.18^e^	14.87 ± 0.53^e^
	12	ND	14.84 ± 0.07^d^	15.25 ± 0.17^d^

*Note*: Different superscripts in the same column on the basis of fruit represent differences between sample groups according to Duncan's test (*p* < .05).

Abbreviations: CH, chitosan‐coated: CHP, Poppy seed extract added chitosan‐coated; NC, non‐coated; ND, analysis not performed because storage was terminated.

Our findings revealed sporadic changes in °Brix values during storage. These variations may be attributed to fluctuating storage conditions, sugar breakdown within the fruit, elevated dry matter content due to water loss, and potential interactions among sample variations (Ghosh et al., 2021; Ozdemir & Gokmen, 2017; Tokatli & Demirdoven, 2020).

In summary, the CHP group's ability to enhance °Brix values in fruits underscores the crucial positive impacts of fruit flavor and quality by elevating sugar content.

In line with existing literature, °Brix values generally increased during storage for all fruits, except pomegranate (Ghosh et al., 2021; Solís‐Contreras et al., 2021; Treviño‐Garza et al., 2017). Applying the coating effectively slows fruit metabolism and respiration rates, minimizing weight loss. Consequently, the concentration of total sugars in the fruits increases, contributing to elevated °Brix values (Riaz et al., 2021). Coatings play a crucial role in preserving the total soluble solids content by limiting the loss of fruit juice and maintaining the fruit's characteristics (Passafiume et al., 2022). Suriati (2021) and Passafiume et al. (2022) reported that coating applications reduce the loss of total soluble solids in fresh‐cut mangoes and help preserve the fruit's physicochemical properties. Passafiume et al. (2021) observed that fresh‐cut pears coated with aloe vera‐based coatings did not significantly increase total soluble solids content during storage and experienced less loss than the control group. Pleșoianu and Nour (2022) reported that in pears with an initial total soluble solids content of 13.5%, the coating application increased this value during storage, while it decreased in the control group. Researchers attributed this increase to postharvest storage's ongoing acid metabolism, where starch and acids are converted into sugars.

### 3.6 Color

The appearance and color of fresh‐cut fruits play a pivotal role in consumer purchasing decisions (Wu et al., 2021). Consequently, the surface color of fruits is intricately linked to consumers' perception of quality, making the impact of applied edible coatings on fruit color a significant parameter. The evolution of L*, a*, and b* values of the fruits within each sample group during storage is outlined in Table 4. According to these results, the color values of the fruit samples varied throughout the storage period.

**TABLE 4:** L*, a*, and b* values of each fruit group in fruit salads.

	Period (day)	L*			a*			b*		
		NC	CH	CHP	NC	CH	CHP	NC	CH	CHP
Pineapple	0	75.39 ± 8.55^a^	78.41 ± 3.07^a^	76.72 ± 2.11^b^	−5.63 ± 0.38^a^	−5.64 ± 1.48^a^	−6.25 ± 0.47^a^	25.80 ± 0.89^a^	26.66 ± 4.27^c^	33.56 ± 2.61^a^
	3	71.84 ± 10.87^b^	73.64 ± 3.48^b^	78.17 ± 4.16^a^	−3.22 ± 1.09^b^	−4.74 ± 0.85^b^	−5.79 ± 0.85^b^	23.26 ± 0.23^c^	27.58 ± 7.16^a^	30.70 ± 4.15^b^
	6	67.64 ± 9.23^c^	71.67 ± 4.17^c^	74.31 ± 1.31^c^	−0.66 ± 0.22^c^	−2.36 ± 1.96^c^	−4.05 ± 1.37^c^	24.48 ± 2.56^b^	26.96 ± 7.58^b^	27.82 ± 3.59^c^
	9	ND	64.98 ± 3.66^d^	64.96 ± 5.56^d^	ND	1.22 ± 2.52^e^	−0.94 ± 1.72^e^	ND	25.03 ± 3.24^d^	25.61 ± 1.10^d^
	12	ND	59.82 ± 1.39^e^	63.94 ± 7.62^e^	ND	2.3 ± 1.50^d^	1.44 ± 1.18^d^	ND	22.55 ± 5.01^e^	23.03 ± 2.16^e^
Apple	0	77.83 ± 1.28^a^	80.09 ± 1.65^a^	77.14 ± 1.66^a^	−6.13 ± 0.61^a^	−7.42 ± 0.21^a^	−8.16 ± 1.21^a^	24.33 ± 0.35^c^	25.34 ± 1.44^e^	28.13 ± 2.67^e^
	3	74.76 ± 3.52^b^	72.28 ± 1.87^b^	69.95 ± 3.96^b^	−4.50 ± 2.28^b^	−2.29 ± 1.13^d^	−2.79 ± 2.19^d^	26.22 ± 2.24^b^	29.78 ± 2.17^c^	33.28 ± 2.02^b^
	6	70.04 ± 4.52^c^	68.44 ± 0.95^c^	66.89 ± 3.33^c^	−0.97 ± 2.14^c^	2.01 ± 1.60^e^	1.80 ± 2.08^e^	27.89 ± 2.78^a^	30.38 ± 1.65^a^	35.29 ± 1.18^a^
	9	ND	68.18 ± 0.75^d^	64.65 ± 1.16^d^	ND	3.65 ± 1.37^c^	5.24 ± 1.02^c^	ND	28.28 ± 0.46^d^	33.25 ± 1.88^c^
	12	ND	64.64 ± 3.34^e^	61.49 ± 1.58^e^	ND	5.64 ± 0.82^b^	6.45 ± 2.02^b^	ND	29.99 ± 1.50^b^	33.14 ± 3.11^d^
Kiwi	0	53.75 ± 3.04^a^	52.50 ± 1.65^a^	48.66 ± 2.28^a^	−12.96 ± 1.2^a^	−11.03 ± 1.3^a^	−9.60 ± 0.40^a^	25.04 ± 1.73^a^	26.99 ± 3.28^a^	23.73 ± 1.15^a^
	3	43.78 ± 3.55^b^	44.28 ± 3.59^b^	37.84 ± 2.71^b^	−9.92 ± 1.78^b^	−7.65 ± 0.77^b^	−5.89 ± 0.24^b^	20.21 ± 2.54^b^	19.54 ± 3.41^b^	14.43 ± 0.36^b^
	6	35.88 ± 1.36^c^	39.10 ± 3.58^c^	36.51 ± 0.96^c^	−8.13 ± 0.76^c^	−4.81 ± 1.55^c^	−3.22 ± 1.10^c^	15.54 ± 1.79^c^	16.34 ± 4.59^c^	13.34 ± 1.92^c^
	9	ND	38.14 ± 3.12^d^	35.47 ± 0.52^e^	ND	−4.04 ± 1.48^d^	−2.54 ± 0.73^d^	ND	15.41 ± 4.15^d^	12.88 ± 0.59^d^
	12	ND	37.87 ± 0.98^e^	36.02 ± 0.13^d^	ND	−2.81 ± 0.51^e^	−1.85 ± 0.32^e^	ND	13.70 ± 0.63^e^	12.30 ± 0.86^e^
Pomegranate	0	29.01 ± 8.55^a^	29.94 ± 3.07^b^	35.34 ± 2.11^a^	20.03 ± 0.38^b^	28.76 ± 1.48^a^	26.24 ± 0.47^c^	7.53 ± 0.89^c^	12.14 ± 4.27^b^	12.06 ± 2.61^c^
	3	25.91 ± 10.87^b^	30.07 ± 3.48^a^	31.31 ± 4.16^b^	22.28 ± 1.09^a^	26.74 ± 0.85^b^	30.13 ± 0.85^a^	8.89 ± 0.23^a^	12.87 ± 7.16^a^	12.22 ± 4.15^a^
	6	18.84 ± 9.23^c^	26.14 ± 4.17^c^	29.47 ± 1.31^c^	18.64 ± 0.22^c^	26.34 ± 1.96^c^	28.03 ± 1.37^b^	8.26 ± 2.56^b^	11.83 ± 7.58^c^	12.21 ± 3.59^b^
	9	ND	25.35 ± 3.66^d^	27.18 ± 5.56^d^	ND	23.63 ± 2.52^d^	25.05 ± 1.72^d^	ND	8.61 ± 3.24^d^	10.96 ± 1.10^d^
	12	ND	22.21 ± 1.39^e^	23.14 ± 7.62^e^	ND	21.78 ± 1.50^e^	22.35 ± 1.18^e^	ND	8.03 ± 5.01^e^	9.26 ± 2.16^e^

*Note*: Different superscripts in the same column on the basis of fruit represent differences between sample groups according to Duncan's test (*p* < .05).

Abbreviations: CH, chitosan‐coated: CHP, poppy seed extract added chitosan‐coated; NC, non‐coated; ND, analysis not performed because storage was terminated.

For pineapple, apple, kiwi, and pomegranate fruits, a decrease in L* values was observed with the increase in storage duration. At the same time, the CH and CHP groups generally had higher L* values than the NC group. This indicates the coating's effectiveness in preserving the fruits' brightness. For the a* values, the NC groups generally had higher a* values, indicating a more pronounced red color in the fruits. However, a* values increased during storage in the CH and CHP groups. This demonstrates the effectiveness of the coatings in preserving and even enhancing the red color of the fruits. As for the b* values, the NC groups typically had higher b* values, signifying that the fruits had yellow–blue color tones. Throughout the storage period, the b* values of the CH and CHP groups were observed to be lower than those of the NC group. This indicates that the coatings effectively preserve the yellow–blue colors of the fruits.

During storage, the L* values consistently declined across all fruits and sample groups. In contrast, the a* value increased for pineapple, apple, and kiwi but decreased for pomegranate. Simultaneously, the b* value decreased in pineapple, kiwi, and pomegranate, while it showed an increase for apple. The decline in the L* values during storage can be attributed to reduced brightness and a more muted appearance resulting from fruit surface dehydration. Typically, a reduction in the L* values and an increase in the a* values during storage signify the initiation of browning, indicating oxidative browning reactions or heightened pigment concentrations. The reduction in the b* value observed in coated samples may be linked to the specific properties and concentration of the polymer integrated into the coating.

In conclusion, the coating formulation enhanced by adding poppy phenolic extract proved more effective in preserving the color values of the fruits. This efficacy stems from the role of poppy phenolic extract in enhancing the fruits' color properties. Poppy phenolics could be pivotal in minimizing color changes and preserving fruit freshness. Notably, similar results have been documented in the literature for apple, kiwi, pineapple, and pomegranate when subjected to edible coating treatments (Farina et al., 2020; Hashemi & Jafarpour, 2021; Jin et al., 2020; Li et al., 2017). Sathiyaseelan et al. (2021) reported the effectiveness of an edible coating composed of chitosan–tea tree oil nanoemulsion and calcium chloride in preserving the color values of bell peppers. Lima et al. (2022) also demonstrated that an apricot phenolic extract‐enhanced coating improved color quality and prevented oxidation in fresh‐cut vegetables. In a study by Pleșoianu and Nour (2022), pectin‐based coatings supplemented with anti‐browning agents effectively restricted adverse changes in color values in fresh‐cut pears, contributing to preserving fruit freshness and visual appeal.

### 3.7 Sensory analysis

The application of edible coatings substantially impacts the sensory attributes of fruits, encompassing texture, aroma, appearance, flavor, and taste. During the first storage day, sensory evaluation of fruit salads resulted in scores ranging from 8 to 9 points for all sample groups, evaluating criteria such as odor, color, taste, texture, and overall acceptability. Notably, there needed to be a discernible distinction in sensory quality between coated and non‐coated fruit salads on the first storage day. Nevertheless, a significant trend emerged where the sensory quality of coated fruit salads exhibited superior preservation, consistently earning relatively higher scores in subsequent storage days compared to non‐coated counterparts.

As the storage period progressed, mold growth in the NC samples led to their exclusion from analysis beyond the 6th day. On this final storage day for NC samples, the 6th day, odor, color, taste, texture, and general acceptability criteria scored at 3, 2, 2, 1, and 1, respectively. In contrast, on the 12th day, the concluding storage day for CH and CHP samples, evaluations for odor, color, taste, texture, and general acceptability were appraised at 4, 5, 4, 3, 4 and 7, 7, 6, 5, 6, respectively.

**FIGURE 3:**
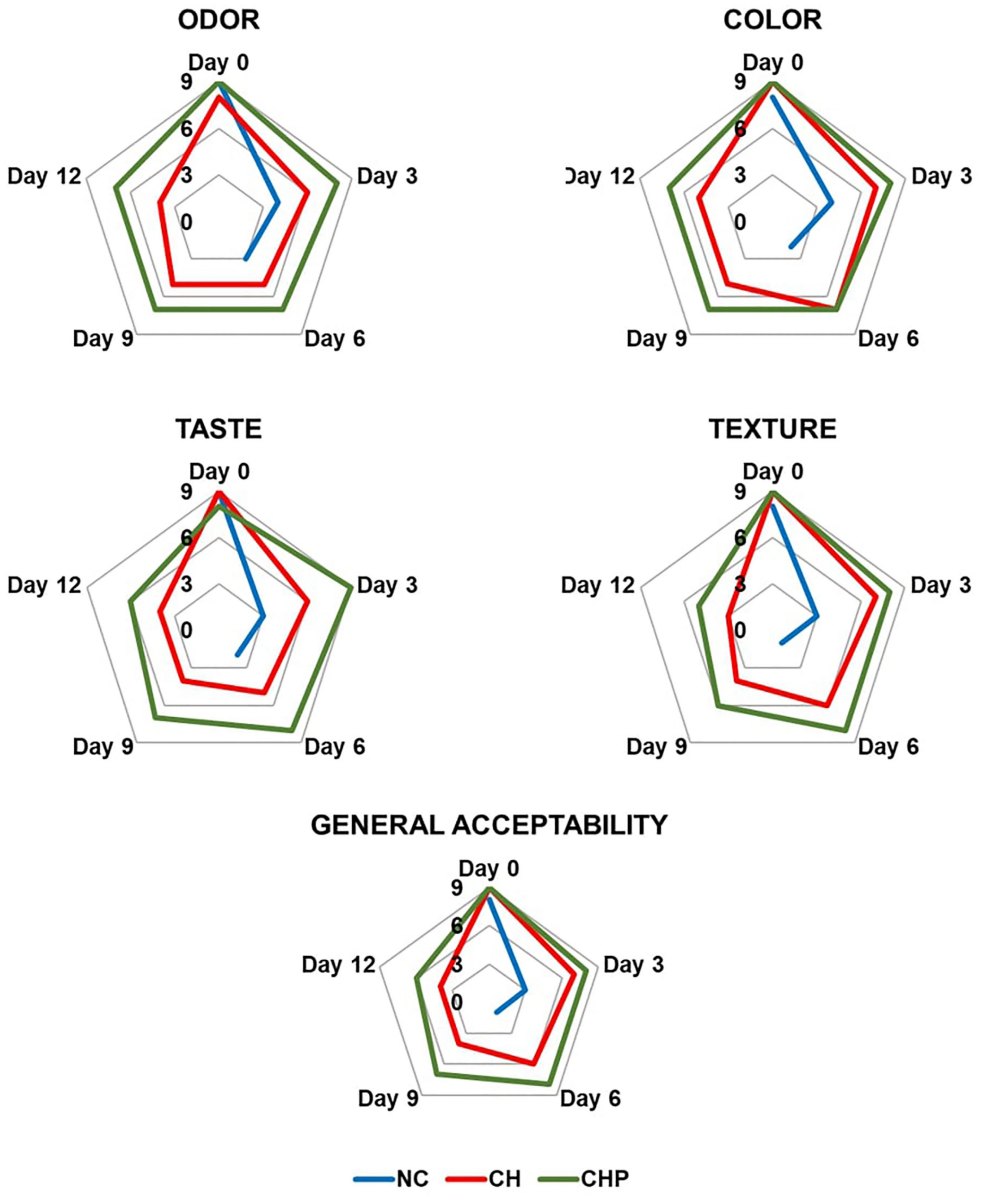
Sensory analysis score (CH, chitosan‐coated; CHP, poppy seed phenolic extract added chitosan‐coated; NC, non‐coated) during storage.

Remarkably, the sensory attributes in coated samples exhibited consistent stability on the 6th, 9th, and 12th storage days, each garnering closely aligned scores. The comprehensive results of all sensory analysis criteria are illustrated in Figure 3.

Throughout the storage period, the color assessment of the NC samples experienced a rapid decline on the 3rd day of storage, followed by a consistent decrease in the subsequent periods. In contrast, in the CH samples, color assessment exhibited stability on the 3rd and 6th days of storage and the 9th and 12th days of storage. Notably, the color attributes of the fruit salads coated with CHP were well preserved on the 6th, 9th, and 12th days of storage.

The observed preservation of color attributes in the CHP samples can be attributed to the inherent gas barrier properties of the chitosan coating, effectively hindering oxidative degradation. Similar findings have been reported in previous studies (Joshi & Awasthi, 2020; Treviño‐Garza et al., 2017), further validating the role of chitosan in safeguarding color characteristics during storage.

Taste scores exhibited a rapid decline in the NC samples but remained relatively stable in both the CH and CHP samples throughout the storage periods. Interestingly, an increase in taste evaluation was observed in the CHP samples between the 0th and 3rd days of storage. This could be attributed to the gradual penetration of the extract used in the coating into the fruits over time, resulting in an intensified taste profile. This finding aligns with previous research that reported taste improvement and decline during storage (Tabassum & Khan, 2020).

Odor values displayed a decrease in the NC samples, exhibited consistent values between the 6th and 9th storage days in the CH samples, and remained stable on the 6th, 9th, and 12th storage days in the CHP samples. Notably, various studies have indicated that chitosan coatings applied to food products do not negatively impact odor characteristics (Solís‐Contreras et al., 2021).

An overall reduction in texture properties was observed across the storage days. This decline in texture could be attributed to several factors, including changes in firmness, soluble solids content, acidity, and ripening of the fruits, which collectively influence the fruit's overall texture (Ghosh et al., 2021).

Concerning the evaluations of general acceptability, it is clear that the application of the CHP offers the most efficient preservation of sensory properties. These results align with similar findings reported by several researchers, emphasizing the role of edible coating applications in maintaining sensory quality attributes in fruits (Fan et al., 2021; Lacivita et al., 2021; Ozdemir & Gokmen, 2017). Sathiyaseelan et al. (2021) reported that an edible coating comprising chitosan–tea tree oil nanoemulsion and calcium chloride positively impacted sensory attributes, such as texture, flavor, and freshness, in bell peppers. Yousuf et al. (2021) stated that edible coatings incorporating essential oils enhanced the sensory characteristics of the products. Alvarez et al. (2021) observed that panelists scored flavor, texture, and appearance similarly to control samples when applied to fresh‐cut apples. Iturralde‐García et al. (2022) reported that coated fresh‐cut fruits were evaluated with scores similar to control samples regarding taste, texture, and appearance. These findings indicate the potential of edible coatings to preserve the sensory quality of fresh‐cut fruits presented to consumers. This supports the development of new technologies that can extend the shelf life of fruits while helping them to maintain crucial attributes like taste and appearance.

Sensory analysis stands as a cornerstone within the food industry, extensively employed in processes related to product development and quality control. The significance of sensory parameters cannot be emphasized enough, as they hold a pivotal role in meeting and exceeding consumer expectations and preferences. Characteristics, such as aroma, texture, appearance, flavor, and overall liking, exert substantial influence over consumers' choices when it comes to purchasing, repurchasing, and gauging their satisfaction with a product. Numerous studies have shed light on the positive impact of edible coatings on the sensory attributes of fresh‐cut fruits. In particular, the application of these coatings to fresh‐cut fruits significantly influences consumer acceptability, as highlighted in the research by Sonti et al. (2002, 2003). Their investigation delved into consumer perceptions of fresh‐cut fruits featuring edible coatings and revealed that a noteworthy 76.5% of consumers would opt for fruits adorned with Food and Drug Administration (FDA)‐approved edible coatings. It is worth noting that consumers tend to shy away from coated fruits if the coating originates from animals. Moreover, households with children are more inclined to embrace the purchase of coated fresh‐cut fruits compared to households without little ones. The collective findings of these studies underscore the paramount importance of enlightening consumers regarding the benefits and advantages that edible coatings bring to fresh‐cut fruits. Evidently, these coatings have the potential to positively influence the sensory attributes and overall quality of the products. In our study, the integration of poppy phenolic extract into the CHP group of fruits yielded consumer perception that consistently garnered high ratings throughout the storage period. This underscores the capacity of edible coatings to preserve the appeal and flavor of fruits throughout their shelf life, all without compromising consumer acceptability.

### 3.8 Microbiological analysis

**FIGURE 4:**
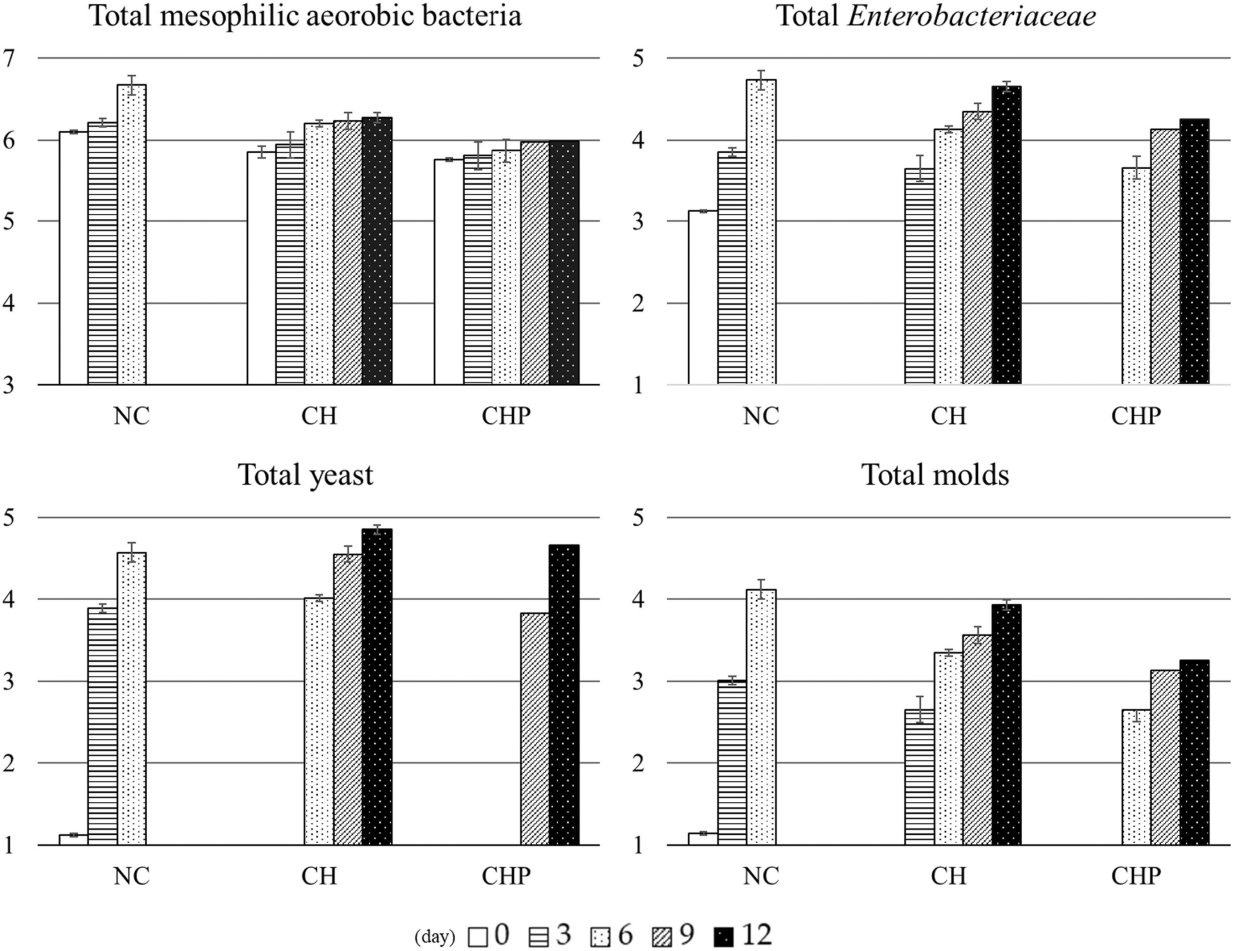
Microorganism loads detected in sample groups (CH, chitosan‐coated; CHP, poppy seed phenolic extract added chitosan‐coated; NC, non‐coated) during storage.

Microbiological analyses of fruits are significant in ensuring quality, hygiene, and food safety (Wu et al., 2021). These analyses are crucial in determining shelf life and consumer acceptance of fresh‐cut fruits. Generally, an acceptable upper limit for fresh‐cut products' total bacterial, mold, and yeast populations ranges between 5 and 6 log CFU/g (Passafiume et al., 2020). Figure 4 illustrates the changes in microbial loads within the sample groups during the storage period. The microbiological analysis results unveil significant variations in total mesophilic aerobic bacteria, total *Enterobacteriaceae*, total yeast, and total molds among different storage groups.

Initial observations of the total mesophilic aerobic bacterial counts reveal similar starting levels across all groups. However, over time, these counts increased within all groups. Notably, the CH and CHP groups displayed a minimal increase compared to the NC group, which, even at the end of the storage period, had yet to reach the bacterial load present in the CH and CHP groups by the 6th day.

Similar trends are noticed in the numbers of total *Enterobacteriaceae*. In the CH group, these bacteria became detectable from the 3rd day onward, whereas in the CHP group, they reached noticeable levels from the 6th day.

When focusing on the values for total yeast and molds, the NC group experienced a considerable increase over time, with visible mold growth detected by the 6th day, leading to the storage termination for this group. In contrast, total yeast appeared in the CH group from the 6th day and in the CHP group from the 9th day. However, even in terms of yeast and molds, these two coated groups failed to reach the values exhibited by the NC group on the 6th day by the end of storage.

These results emphasize that coatings containing chitosan, especially the CHP formulation, effectively enhance the microbial quality of fruit salads. The underlying mechanism for this efficacy can be attributed to the antimicrobial properties of the poppy seed phenolic extract incorporated into the formulation. The ability of the poppy seed phenolic extract to inhibit the growth of bacteria, yeast, and molds may effectively curtail the proliferation of microorganisms, thereby prolonging the shelf life of fruit salads.

The higher mold and yeast values detected in the NC samples can be attributed to the lower pH values of untreated fruit salads. The presence of chitosan within the coating matrix may effectively limit this microbial development, as chitosan's inherent alkaline properties contribute to the protection of the product. In contrast, prior reports suggest that chitosan coatings can inhibit bacterial growth by forming a protective layer on the fruit's surface. Our findings are consistent with existing literature data (Basaglia et al., 2021; Rodríguez et al., 2021; Wu et al., 2021). A noteworthy observation emerges when we compare the CH and CHP samples: the microbial load in the CHP samples was lower. This outcome suggests that the extract derived from the poppy seed contains phenolic components and antimicrobial agents. In summary, the results from microbial analyses underscore the significance of edible coatings in mitigating microbial growth and highlight their role in enhancing the safety and quality of fresh‐cut fruits.

## 4 DISCUSSION

The present study offers a comprehensive assessment of the influence of edible coatings on the quality and shelf life of fresh‐cut fruit salads by integrating various quality parameters, including weight loss, pH, titratable acidity, browning index, decay percentage, total water‐soluble solids content, color attributes, sensory analysis, and microbiological analysis. The multifaceted relationships among these parameters provide valuable insights into the effectiveness of the applied coatings, particularly the CHP formulation.

Weight loss is a critical parameter affecting the quality of fresh‐cut fruit salads during storage. In our study, all groups experienced a progressive increase in weight loss; however, the difference between the NC and coated groups, especially the CHP groups, was noteworthy. The CHP group exhibited the lowest decay percentage, highlighting the superior protective capacity of the applied coating. This observation underscores the significant role of edible coatings, particularly those enriched with poppy seed phenolic extract, in mitigating weight loss and extending the shelf life of fresh‐cut fruit salads.

Changes in pH and titratable acidity levels indicated the influence of coatings on the metabolic activities of the fruit salads. The decline in pH values of the NC samples suggested the potential of coatings, particularly CHP, in maintaining a favorable pH profile. The complex behavior of titratable acidity, with a reduction in the NC group but an increment in the CH and CHP groups, further illustrates the effect of coatings in preserving the acidic and organoleptic qualities of the fresh‐cut fruit salads.

Browning, as a pivotal quality parameter, was effectively managed by the coatings. The higher browning index in the NC group underlines the protective role of coatings against undesirable color changes driven by oxidative browning reactions. In contrast, the CHP group exhibited the lowest browning index, indicating the potential of the poppy seed phenolic extract to counteract browning reactions with its antioxidants.

The evolution of °Brix values pointed to the role of coatings in mitigating water loss and concentrating sugar content in the coated groups. However, the irregularities in °Brix values could be attributed to complex interactions involving water loss, sugar breakdown, and sample variability, emphasizing the coatings' role in preserving the fruit salads' sugar content and contributing to their sensory quality.

Color attribute changes, as reflected in L*, a*, and b* values, demonstrated the complexity of preserving the visual appeal of fresh‐cut fruit salads. The observed reduction in L* values, increased a* values, and unique behavior of pomegranate suggested that coatings could mitigate undesirable color shifts.

Sensory evaluation consistently favored the coated fruit salads, particularly the CHP formulation, preserving taste, odor, color, texture, and overall acceptability. These sensory results highlight the benefits of using edible coatings in ensuring that the fruit salads remain appealing and flavorful throughout their shelf life.

Microbiological analysis revealed that coatings, especially the CHP formulation, effectively mitigated microbial growth. The antimicrobial potential of the incorporated poppy seed phenolic extract was evident in the reduced microbial load in the coated samples, further emphasizing the multifaceted role of coatings in preserving the safety of fresh‐cut fruit salads.

The findings revealed several interrelated parameters contributing to these products' overall quality and preservation. Notably, a low initial pH was associated with increased microbial growth, as observed in the NC group, where a notable decline in pH coincided with higher microbial loads and accelerated decay. In contrast, higher °Brix values in the coated groups were linked to reduced decay, indicating that coatings mitigate water loss and concentrate the sugar content. The elevated pH levels in the CHP group suggest that the added phenolic extract potentially played a role in maintaining a favorable pH profile, contributing to enhanced product quality. Moreover, the results demonstrated that coatings, especially the CHP formulation, were influential in preserving taste, odor, color, texture, and overall acceptability, which aligns with the lower microbial loads observed in these groups.

Adhering to strict legal regulations is vital for ensuring the safety of edible coatings and safeguarding consumer well‐being. Edible coatings, thin layers on food surfaces safely consumed as part of the product, must comply with the U.S. Federal Food, Drug, and Cosmetic Act. They are classified as generally recognized as safe (GRAS) and used in appropriate quantities. European Directive and U.S. regulation categorize edible coatings as various food‐related entities. Coatings, integral to the edible portion, must meet food content guidelines. Functional additives and film‐forming components must be food‐grade and non‐toxic. Application rates adhere to the country's legislation. The European Directive permits specific components in edible coatings. In 1998, it expanded to include additional substances. U.S. regulations allow supplementary food additives for protective coatings on fresh produce. Major legal regulations come from the FDA, European Union standards, and Codex Alimentarius. The FDA necessitates GRAS or regulated food additives in formulations, with usage restricted. Labels must indicate functional additives in edible films and coatings. In Europe, food additives should be clearly labeled. Another critical aspect is allergens, as many edible films and coatings may contain allergenic components. Milk, soybeans, fish, peanuts, nuts, and wheat are common allergens in these coatings. Products with allergenic coatings should have clear labels to inform consumers. In summary, strict adherence to legal regulations is essential for edible coating safety. Clear labeling is crucial to inform consumers about their presence, including potential allergenic components (Dhall, 2013; FDA, 2006; Maringgal et al., 2020; Rojas‐Graü et al., 2009; Vargas et al., 2008).

Our findings align with previous research and underscore the positive impact of coatings on various quality parameters. The results highlight the potential of edible coatings, especially those enriched with poppy seed phenolic extract, in enhancing the shelf life and quality of fresh‐cut fruit salads.

## 5 CONCLUSIONS

Implementing a chitosan‐based edible coating fortified with phenolic extract derived from poppy seeds showcased substantial enhancements in the quality characteristics of fresh‐cut fruit salads. This innovative formulation exhibited commendable impacts on multiple facets, including weight loss, pH, titratable acidity, browning, water‐soluble dry matter, color, and decay, throughout the storage period. Significantly, the coating's efficacy transcended mere physicochemical attributes, encompassing sensory and microbiological dimensions. The chitosan‐based coating, bolstered by the inclusion of poppy seed phenolic extract, emerged as a pivotal factor in extending the shelf life of fresh‐cut fruit salads. Simultaneously, this coating strategy diligently retained the sensory allure and garnered high levels of consumer approval. This study underscores the considerable potential of such coatings as a transformative tool in elevating the quality and safety of fresh produce. The findings contribute to the ongoing advancement of sustainable and effective methods for preserving the integrity of food products, thereby advancing the pursuit of a more resilient and environmentally conscious food industry.

## 6 FUTURE PROSPECTS

This study reveals exciting avenues for future research, primarily focusing on harnessing the potential of poppy seed phenolic extracts and elevating the quality of fresh‐cut fruit salads. To pave the way for further investigations, we suggest the following directions: First, the exploration of poppy seed phenolic extracts can delve deeper into the properties and applications of these compounds, including identifying specific phenolic compounds within the extract and understanding their individual effects on microbial inhibition, antioxidant properties, and quality preservation. Second, future research can seek to develop tailored edible coating formulations enriched with various natural extracts, such as those from spices, herbs, or fruits, to enhance the preservation of fresh‐cut fruit salads. These studies may examine different extracts' compatibility and synergistic effects when combined with chitosan‐based coatings. Third, developing sustainable packaging materials complementing edible coatings offers excellent potential. Eco‐friendly and biodegradable packaging materials, when combined with coatings enriched with phenolic extracts, can significantly reduce food waste and enhance product safety. Additionally, research into consumer preferences and market dynamics is essential. Insights from consumer studies can guide the development of products that align with consumer needs and preferences, ultimately shaping the future of fresh‐cut fruit salads. Assessing the nutritional changes that occur during storage and evaluating the impact of edible coatings on nutrient retention is another important avenue for future research. This information is vital for ensuring that extended shelf life does not compromise the nutritional value of the products. Lastly, scaling up the application of these coatings to commercial production and collaborating with the food industry are pivotal steps for broadening the impact of innovative coating formulations and making extended shelf life and quality preservation accessible to a broader consumer base. In summary, using plant‐based phenolic extracts promises to revolutionize the fresh‐cut produce industry, meet the growing demand for healthy and convenient food choices, and enhance product safety and quality.

## AUTHOR CONTRIBUTIONS

**Bahar Demircan:** Conceptualization (equal); data curation (equal); formal analysis (equal); methodology (equal); resources (equal); software (equal); validation (equal); visualization (equal); writing – original draft (equal). **Yakup Sedat Velioglu:** Conceptualization (equal); funding acquisition (equal); investigation (equal); methodology (equal); project administration (equal); supervision (equal); writing – review and editing (equal).

## FUNDING INFORMATION

This research was funded by Ankara University Scientific Research Projects Coordination Unit, grant number 2200443002.

## CONFLICT OF INTEREST STATEMENT

The authors declare no conflict of interest.

## Data Availability

Not applicable.
